# The impact of inflation on medicine prices in Nigeria: a comparative analysis of public and private pharmacies

**DOI:** 10.11604/pamj.2024.49.56.45188

**Published:** 2024-10-25

**Authors:** Kelechi Julian Uzor, Modupe Lydia Olarewaju, Temitope Kemi Asishana, Amana Effiong, Anderson Uchenna Amaechi

**Affiliations:** 1 Imperial College Business School, London, United Kingdom,; 2 University of Ilorin Teaching Hospital, Ilorin, Nigeria,; 3 Catholic Caritas Foundation of Nigeria, Abuja, Nigeria,; 4 University of Geneva, Geneva, Switzerland

**Keywords:** Inflation, access to medicines, health financing, health insurance

## Abstract

Access to medicines remains a significant challenge in low-and middle-income countries. In Nigeria, access to medicines is particularly difficult due to a mix of factors such as high out-of-pocket expenditure, inequitable distribution of healthcare facilities, and health workforce brain drain. Recently, this has been further exacerbated by adverse macroeconomic factors such as high inflation and currency devaluation due to weak international trade. In this paper, the authors examine the rising cost of medicines in Nigeria's private and public pharmacies over a period of 12 months from March 2023 to February 2024. By conducting market research and a quantitative analysis of price changes across three drug classes, the study reveals consistent price increases for medicines sold in the public and private sector; with the most increase occurring in the private sector. The findings highlight that these price pressures, in addition to limiting access to medicines, can affect healthcare businesses adversely, leading to more job losses and worsening inequality. The paper concludes with a discussion of the necessity of robust social protection policies to mitigate the financial burden on patients.

## Introduction

In recent times, poor access to medicines has been exacerbated by rising commodity prices attributable to the country's weak macroeconomic outlook [1]. While most Nigerian commentaries on inflation - broadly defined as “the rate of increase in prices over a given period of time” - focus on food inflation, this paper sheds light on the rising cost of medicines in private and public pharmacies [2,3].

**Figure 1:**
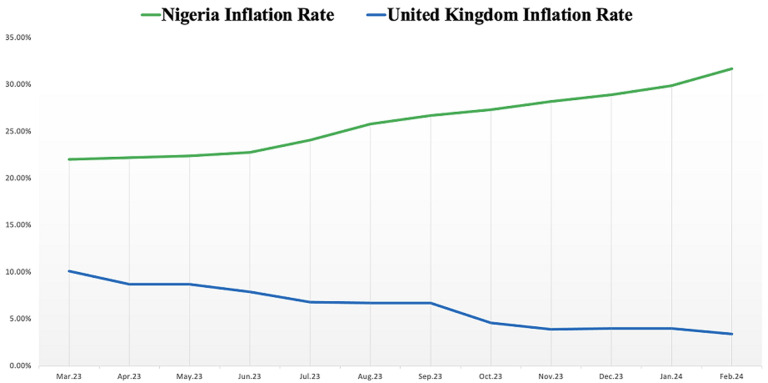
inflation rate in Nigeria and the United Kingdom, from the period between April 2023 and March 2024

The impact of pricing on access to medicines is significant, particularly in Nigeria, where out-of-pocket healthcare costs are disproportionately high and health insurance coverage is limited [4,5]. Following the impact of the COVID-19 pandemic on the global economy, many countries have rebounded from the initial shock, with some countries recording economic growth comparable to pre-pandemic levels [6]. However, this does not hold true for many African countries. For instance, while the inflation rate in the United Kingdom (popularly referred to as the cost-of-living crisis) has fallen from 8.7% in April 2023 to 3.2% in March 2024, Nigeria's inflation has risen from 22.22% to 33.20% over the same timeframe (Figure 1), surpassing forecasts from the Central Bank of Nigeria and independent monetary policy analysts [7,8]. Recent evidence suggests that inflationary pressures are negatively impacting the operating environment in Nigeria, resulting in the closure of healthcare businesses and pharmaceutical companies [9]. If the current trend continues, patients will likely face increasing challenges in affording healthcare services.

## Case study

### Methods

The authors conducted market research and analysis of sales data from a public and private pharmacy between March 2023 and February 2024. Both pharmacies are located in North-Central-Nigeria; the public pharmacy is a federally funded tertiary hospital while the private pharmacy is a sole proprietorship with significant sales in volume. For each pharmacy, the selling prices of three classes of medication (antibiotics, anti-cancer medicines, and anti-hypertensives) were analysed over a period of 12 months as defined above. These prices were then compared between the two groups (private and public) to identify the key differences in pricing between the two groups. The specific names of both centres have been anonymised in the report to protect sensitive market and competitor information.

The study ensured rigorous data collection and validation processes, including cross-referencing prices with official records from Nigeria's National Bureau of Statistics (NBS) to enhance the reliability of the findings. The authors also conducted follow-up interviews with key stakeholders to gain deeper insights into the underlying factors influencing price changes. A statistical analysis of proportions was conducted on MS Excel, with price changes reported per quarter. In the sections below on results and discussions, we describe these findings and highlight the role of evidence generation in developing context-driven public policies for health markets.

## Results

**Figure 2:**
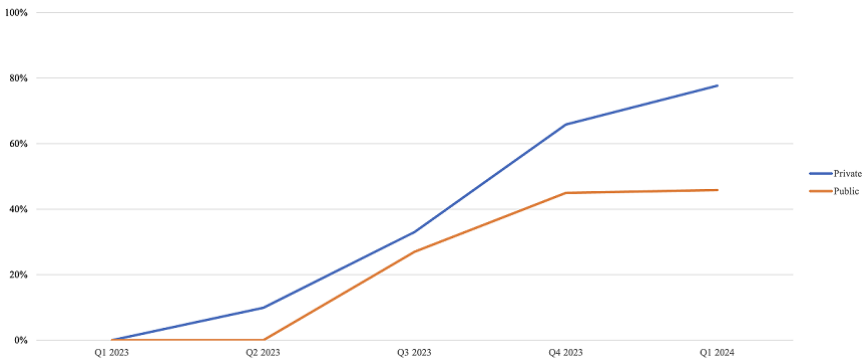
quarterly percent change in the retail price of antibiotics from Q1 2023

**Private pharmacies respond swiftly to market pressures:** this is not unexpected, given the profit-driven nature of private enterprises. In the antibiotics market, the authors analysed five classes of drugs used in treating infectious diseases and found that between Q1 2023 and Q1 2024, prices had changed by about 78% in the private sector compared to 46% in the public sector. As seen in Figure 2, while prices of antibiotics remained relatively stable in the public sector between Q1 and Q2 of 2023, there was a 10% change in the cost of these products in the private sector during the same time frame. While it could be argued that patients might prefer to procure medicines cheaply from public pharmacies, issues such as stock-outs are widespread in the public sector, thereby limiting access to medicines for patients [9].

**Price changes in anti-cancer medicines were higher in public pharmacies:** between Q1 of 2023 and Q1 of 2024, prices of anti-cancer medicines increased by 75% in the public sector pharmacy compared to just 33% in the private sector pharmacy. Key informant interviews of management staff conducted as part of the analysis revealed that anti-cancer medicines were restocked regularly because of the high demand for specialist oncology services in the hospital's cancer centre. This explains, in part, the frequent price increases in response to prevailing market conditions.

**Business models are important drivers of pricing in healthcare operations:** the business model of public sector pharmacies in Nigeria has been deliberately designed to increase access to medicines, with little emphasis on profitability. This has a direct impact on the ability of the pharmacies to re-stock commodities - leading to more stock-outs in public sector pharmacies when compared to privately-run pharmacies. In terms of operations, public sector pharmacies are on average, larger procurers of medicines, leveraging economies of scale to buy higher volumes of medicines at reduced prices. In this study, the average retail price of anti-hypertensives increased by only 24% over a period of one year in the public pharmacy compared to 134% in the private sector.

## Discussion

Findings from the market research show how prices of essential medicines increased over a short timeframe in response to macro-economic forces. While this study cannot be generalised given the limited sample size of pharmacies surveyed, there is evidence that the inflationary pressures on the Nigerian economy are not limited to price changes in the cost of food and fast-moving consumer goods. Additionally, this study did not examine the relationship between price changes and the demand for health services, it is highly likely that patients would be unable to afford medicines as prices increased. In lieu of the above, how should health systems respond to rising out-of-pocket costs for medicines? Below, we propose three ideas that cut across both the public and private sectors.

First, expanding health insurance coverage to millions of people who are currently uninsured will reduce the financial risk associated with rising drug prices. The 2022 National Health Insurance Authority Act which aims to expand health insurance coverage to over 83 million poor people is expected to reduce financial risks associated with rising drug prices [10]. While increasing drug prices may increase the cost of health services and health insurance premiums, a well-managed health insurance system - inclusive of social and community health insurance schemes - will create a balance between the supply of health services and the resources required to fulfil the demand for the services. Ultimately, this will increase the resource pool required to scale health system investments in Nigeria.

Second, a significant driver of the increased prices of drugs is Nigeria's import dependence and a volatile foreign exchange. Increasing investments in Nigeria's health system and the ability of the country's population to fulfil the demand for health services through a robust insurance system will stimulate investments in local manufacturing of drugs and medical products. For policymakers, this would involve strengthening Nigeria's regulatory environment and ensuring overall improvement in the ease of doing business.

Third, in many public hospitals in Nigeria, medicines stock-out remains a significant issue and is likely to worsen as the purchasing power of healthcare businesses and consumers depletes from higher prices and a devalued currency. Ensuring the consistent supply of drugs through increased investments in local drug manufacturing and a robust demand through a well-funded health insurance system is essential in building a sustainable healthcare ecosystem. This should be completed through data-driven inventory platforms - thereby ensuring efficient stock management of health products in hospitals and pharmacies.

## Conclusion

The study underscores the critical impact of inflation on drug prices in Nigeria, with significant disparities between the private and public sectors. The rapid price increases in private pharmacies, driven by profit motives and economic pressures, contrast with the more stable but often stock-out-prone public pharmacies. The unique challenges in the pricing of anti-cancer medicines in the public sector further illustrate the complexities of healthcare provision in an inflationary environment. To ensure equitable access to essential medicines, especially for managing chronic conditions like hypertension, it is imperative to develop comprehensive social protection policies and enhance the efficiency and capacity of public pharmacies. This approach will help mitigate the adverse effects of economic volatility on healthcare accessibility and affordability in Nigeria.
